# The efficacy of home-based virtual reality exposure therapy as an add-on to behavioral therapy for children with selective mutism: Protocol for a single-case experimental design

**DOI:** 10.1016/j.conctc.2026.101602

**Published:** 2026-01-16

**Authors:** Wendy van Vlerken, Jeroen S. Legerstee, Robert G. Belleman, Samantha Bouwmeester, Lynn F. Meester, Annelot Roorda, Ramón J.L. Lindauer, Elisabeth M.W.J. Utens, Luuk Stapersma

**Affiliations:** a Amsterdam UMC, University of Amsterdam, Department of Child and Adolescent Psychiatry, Amsterdam Public Health, the Netherlands; b Academic Center for Child and Adolescent Psychiatry Levvel, Amsterdam, the Netherlands; c Research Institute of Child Development and Education, University of Amsterdam, the Netherlands; d Informatics Institute, University of Amsterdam, the Netherlands; e Out of the Boxplot, Rotterdam, the Netherlands

**Keywords:** Virtual reality, Exposure therapy, Children, Selective mutism, Anxiety disorder

## Abstract

**Background:**

Selective Mutism (SM) is a rare childhood anxiety disorder characterized by an inability to speak in specific social situations, despite speaking freely in others. School is typically the environment where the disorder manifests itself most clearly. Children with SM often have difficulty generalizing speaking across different social situations. Parents and teachers often struggle to practice speaking with the child outside of therapy due to practical reasons and time constraints. Virtual Reality Exposure Therapy (VRET) at home has potential to enhance the treatment of SM by providing an alternative and engaging method for delivering exercises that support behavioral treatment.

**Methods:**

This paper describes the development of the VRET application Speaking at School-VR and the methodology of the single case experimental design (SCED) to evaluate the feasibility and preliminary effects of the behavioral treatment at school, complemented with VRET at home, for children with SM, aged 4–13. A secondary aim is to evaluate the feasibility and adherence of home-based VRET based on qualitative information to capture participants’ experiences. A total of 15 children with SM and their families will be enrolled in the study to examine the potential effectiveness of the combined treatment. The treatment consists of 10 behavioral steps, each accompanied by complementary VRET exercises.

**Conclusion:**

This is the first study to examine the feasibility and the potential effectiveness of home-delivered VRET as an adjunct to behavioral treatment for children with SM. The addition of VRET to behavioral treatment has the potential to enhance the generalization and transfer of skills across different situations in a facilitative and engaging way.

## Highlights

• First study applying home delivered VRET for children with selective mutism.
• Immersive VR is used to support gradual exposure at home settings.
• Each step of the treatment protocol has corresponding VRET exercises.
• Study will assess feasibility, usability, and acceptability of the protocol.

## 1 Background

Selective Mutism (SM) is a rare childhood anxiety disorder characterized by the absence of speech in specific social situations (e.g. at school), despite being able to speak freely in other situations (e.g. at home). SM symptoms also manifest in playground interactions, social communication with peers or family members, and extracurricular activities. Prevalence of the disorder is estimated from 0.2 % up to 1.6 % among the general population [[1], [2], [3], [4]]. This prevalence tends to be slightly higher among immigrant children and children with speech- and language delays. The disorder often goes unnoticed until the child transitions to primary school, as this is typically the first setting where children are required to engage independently with unfamiliar people. Additionally, increased social demands and academic expectations at school make the symptoms more apparent [5]. As a result of not speaking, children with SM are at risk of missing out on learning and social activities [6], and if left untreated, the symptoms might become chronic. This may increase the risk of both social- and academic stagnation [2,7]. Early detection and intervention are pivotal as they might help to mitigate potential delays and promote increased well-being.

Cognitive Behavioral Therapy (CBT), is an evidence-based and widely used treatment for SM [8]. Given the developmental level of young children with SM and their limited verbal abilities, CBT interventions tend to emphasize behavioral approaches, with limited use of cognitive components [9,10]. Treatment for SM is primarily based on behavioral therapeutic principles, with a focus on gradually increasing verbal behavior through carefully structured exposure to speaking situations [11]. Notably, school-based interventions have proven especially effective in addressing the context-dependent nature of the anxiety experienced by children with SM [9]. However, despite these positive outcomes, long-term follow-up studies reveal that former patients often continue to experience communication difficulties and have a higher risk of comorbid psychiatric conditions [4,12]. This underscores the need to optimize existing interventions to support the generalization of speech consolidate treatment gains, which could help mitigate long-term difficulties.

CBT has demonstrated its effectiveness in treating SM, however the therapeutic process can be challenging as it requires knowledge of the specific population and relies on specialized techniques (e.g., defocused communication and finely graded exposure) and must be implemented across home and school contexts, requiring consistent collaboration between therapists, parents, and teachers [10]. Additionally, it often takes time to build rapport with the child, and it may take additional time for the child to feel comfortable speaking in front of a therapist and to generalize speaking across different contexts [13]. To support generalization, parents and teachers are encouraged to continue practicing speaking outside of therapy to reinforce treatment gains [14]. However, establishing effective collaboration between parents and schools can be challenging, as teachers in primary education often have competing responsibilities and limited time. At the same time, it can be difficult for parents to create meaningful possibilities for exposure, particularly when the child already speaks freely at home. Although CBT is effective, the generalization of speech beyond therapy sessions remains challenging. Virtual reality exposure therapy (VRET) may help to address this limitation by providing safe, simulated exposure contexts. Delivered in a home setting, VRET has the potential to address these challenges by providing structured, controlled speaking scenarios in addition to traditional therapy sessions.

A meta-analysis shows that VRET has established itself as an effective treatment alternative, next to real-life and imaginary exposure, for treating anxiety disorders [15]. The use of VRET offers unique advantages over traditional exposure. Virtual reality (VR) can be used to create a safe, realistic and immersive simulation of a stressful situation [16] and can enhance treatment motivation by amplifying the gamified elements of the treatment [17]. The use of VRET allows for more flexibility during the treatment since exposure parameters can be adjusted depending on the individualized needs of the client [18]. Considering exposure learning theory, flexibility is especially important as repeated and graded exposure to realistic but non-threatening speaking situations facilitates habituation, which in turn reduces the child's anxiety response. In addition, VRET promotes the generalization of speaking by facilitating practice across several virtual environments that resemble real-life school environments.

An additional advantage is that VRET is not bound to a location and can therefore be implemented at home without the guidance of a therapist [16,19]. The implementation of VRET at home provides the child with a safe and familiar setting to practice speaking without the pressure of social evaluation. By creating positive speaking experiences in a virtual school environment, the child can gradually build confidence and begin to transfer these skills to more challenging settings, such as the therapy room, the classroom, and other social situations. In this way, VRET directly targets the core mechanisms of SM—context-dependent speech inhibition and limited cross-situational generalization and bridge the gap between speaking at home to speaking at school. Thus far, only one study has examined the feasibility of VRET as an adjunct for CBT children with SM [20]. Children participated in six therapist-led semi-immersive VRET sessions, displayed on a 42-inch large LCD TV screen, after receiving eight CBT and/or anxiety management sessions. The children's overall functioning improved, and parents reported that the VRET was an acceptable treatment for their child. In contrast to the current study, the use of a TV screen might limit the level of realism and the feeling of presence and immersion [21], which in turn might reduce the efficacy of the intervention**.** In addition, the prior study introduced VRET only after children had completed CBT, which restricts conclusions about the use of VRET integrated within CBT. In contrast, our study evaluates parent-led VRET embedded within ongoing school-based CBT. Based on the principles of exposure learning therapy, the integration of VRET within the treatment offers a distinct advantage as it provides graded, realistic and non-threatening speaking situations that may facilitate habituation and support the generalization of speech across different context. The integration of VRET at home, highlights both the novelty and necessity of the present research. To our knowledge our study is the first to apply parent-led VRET as an adjunct behavioral treatment that is given at school. The aims of this study are 1) to develop a VRET application ‘Speaking at school’ that aligns with the CBT treatment protocol for SM, 2) to examine the efficacy of a combined CBT intervention ‘Speaking at school, a matter of doing’ with home-delivered VRET as an adjunct, by means of Single Case Experimental Design (SCED), and 3) to explore which factors contribute or hinder the use of VRET at home and identify which clients benefit most from the intervention. For this purpose, we included relevant measures to assess child factors (such as immersive tendencies, communication) and familial factors (such as parental behavioral and emotional problems, family accommodation and parenting burden).

## 2 Design and methods

**Fig. 1:**
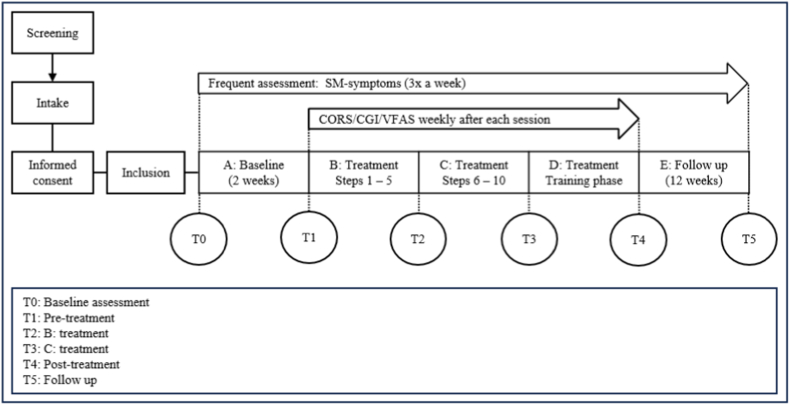
Study design.

**Study Design:** This study is a single-case experimental design (SCED) carried out in a specialized academic center for child and adolescent psychiatry (Levvel, Amsterdam, The Netherlands). In a SCED, statistical power is generated through frequent and repeated measurements of the primary outcome over time to monitor within-subject changes [22]. This makes a SCED highly suitable for measuring the treatment effect of individualized treatments (such as children with SM). Due to their adaptability and statistical rigor, SCEDs can be used for innovative treatments in order to generate initial evidence before moving on to more traditional group-based designs [22,23]. This study employs a five-phase design: baseline (A: 2 weeks, no treatment), three individualized treatment phases. The first treatment phase (B: protocol step 1–5, treatment) requires the child to respond to the teacher by producing sounds. Treatment phase two requires the child to respond verbally to teacher (C: protocol step 6–10, treatment). During the last phase of the treatment focuses on the intensification of verbal communication to solidify treatment gains (D: training phase, treatment) and follow-up phase (E: 12 weeks, no treatment). Previous research in our center concluded that the average treatment duration is on average 25 weeks (see Fig. 1).

Experience sampling method (ESM) assessments (see Paragraph 2.3.1 and Appendices A and B) are used to quantify SM symptoms. These assessments are conducted thrice weekly via the M-path smartphone application [24] by parents and teachers. Considering the average treatment length of 25 weeks, yielding approximately 117 time points across all phases. To ensure adherence and feasibility, the study design was developed balancing a valid number of frequent assessments (after consulting a statistics expert, SB) with the burden for participants, based on ample experience of our research group in conducting ESM studies.

ESM measures are supplemented by comprehensive assessments at key time points. The comprehensive assessment consists of a standardized diagnostic measure of the primary outcome (i.e. SM symptoms), secondary outcomes (i.e. emotional and behavioral problems, anxiety, parenting burden, family accommodation and VR evaluation) as well as relevant predictors, covariates, and potential confounding factors (i.e. parental behavioral and emotional problems, parenting style, immersive tendencies and communication). Fig. 1 provides an overview of the study procedures and assessments conducted at each time point.

The baseline assessment [T0] includes both primary and secondary outcomes, and potential confounding factors, as reported by parents, teachers, and children. At the start of each study phase [T1], [T2], and [T3] parents and teachers report on the *primary outcome* only. Post-treatment [T4] and follow-up [T5] assessments are similar to the baseline assessment, measuring only the primary and secondary outcomes across all informants. Due to the individualized treatment approach, clinical assessments are standardized by therapeutic progress rather than chronological time, resulting in variable intervals between assessments across participants.

**Inclusion criteria:** Children are eligible if they attend primary school, are 4–13 years old, and are referred to our center for diagnosis and treatment for SM between January 2025 until December 2026. Psychiatric comorbidity is permitted, as long as SM is the primary treatment target. Medication use is allowed, provided it has been stable for four weeks prior to participation and remains unchanged throughout treatment.

**Exclusion criteria:** Potential participants that meet any of the following criteria will be excluded from participation in this study: Parents have insufficient mastery of the Dutch language, parents or the child have an estimated IQ < 85, the child needs inpatient treatment, children that received protocolled behavioral therapy for SM in the previous 12 months, children with a (medical) condition that is a contraindication for the use of VR (e.g. epilepsy, claustrophobia, facial deformities that prohibit the use of a VR headset, motion sickness and panic attacks).

### 2.1 Patient recruitment and procedure

The research protocol was approved by the Medical Research Ethics Committee of Amsterdam UMC (ID: NL86645.018.24). Eligible children and their parents will be informed about the study during the intake assessment. Those interested in participation will be contacted by the researcher and will receive written information about the study. A follow-up appointment will be scheduled to give participants the opportunity to ask questions and for the child to try on the VR headset. Informed consent will be obtained from both parents. Children aged 12 and 13 will also be asked to provide written informed consent. After signed informed consent, participants are enrolled in the study. See Fig. 1 for study procedures.

Upon completion of the baseline assessment, the researcher will visit families to set up the VR headset (Meta Quest 3 [25], supplemented with an additional head strap to enhance comfort) and provide parents with instructions for use. Throughout the study period the researcher will pro-actively contact participants once a month to foster adherence to the VRET exercises and to ensure proper usage. Parents are encouraged to contact the researcher if they encounter issues in between the scheduled appointments. After the post-treatment assessment, participants are invited to an interview to share their experiences working with the Speaking at School-VR.

### 2.2 Intervention

#### 2.2.1 Behavioral treatment

Children receive the behavioral treatment protocol "Speaking at School, A Matter of Doing" (in Dutch: ‘*Praten op school, een kwestie van doen’*) [26]. The protocol is specifically designed to treat SM in primary school-aged children. The therapy is delivered at school, as SM symptoms are typically most prominent in that environment. The exercises are interactive and include game-like elements. Treatment begins in a separate room at school, and as therapy progresses, the child and therapist gradually move towards the classroom.

The treatment includes 10 hierarchical exposure steps, in which the child gradually learns to produce sounds and practices speaking at school (see Table 1). During the first five steps of individual treatment, the child is required to give a non-verbal response in class. In the final five steps, the child must respond verbally to the teacher and gradually begin speaking in class. Once the child can speak in class, the treatment is followed by a training phase to consolidate the results. Treatment concludes when the child consistently gives verbal responses in class and can participate in classroom discussions. After each session, the therapist provides the teacher with instructions to maintain treatment progress in the classroom. On average, the treatment lasts 25 weeks.

**Table 1:** Overview of the exercises of the Speaking at School-VR.

	Name	Description	Environment
Step 1	Foosball	Try to score a goal by blowing a ball across the foosball table.	Playground
Step 2	Ring the bell	Blow a lever to move the weight upward to ring the bell.	Playground
Step 3	Animal sounds	Imitate animal sounds	Corridor
Step 4	Sounds and words	Repeat phonemes and repeat word that is formed.	Corridor
Step 5	Repeating words	Repeat the words that is shown on the picture.	Corridor
Step 6	Naming pictures	Name the picture that is shown.	Classroom
Step 7	Combining words	Identify an object, state the color, and combine the object and the color.	Classroom
Step 8	Making sentences	Make a sentence of the pictures that are shown.	Classroom
Step 9	Finish the sentence	Finish a partially completed sentence by adding the missing ending. The goal is to construct and say the entire sentence.	Classroom
Step 10	Fun with sentences	Describe a picture using multiple sentences.	Classroom

#### 2.2.2 Development and implementation of speaking at School-VR

The application Speaking at School-VR is a collaboration between the Visualisation Lab of the Informatics Institute, University of Amsterdam,^1^ and psychologists and researchers from Levvel and the Amsterdam UMC. The application was developed as an adjunct to the CBT treatment ‘Speaking at School, a Matter of Doing’, aiming to facilitate generalization of treatment results.

**Fig. 2:**
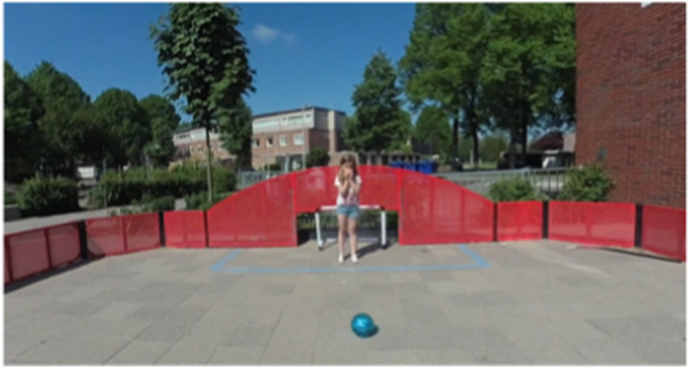
Prototype playground.

**Fig. 3:**
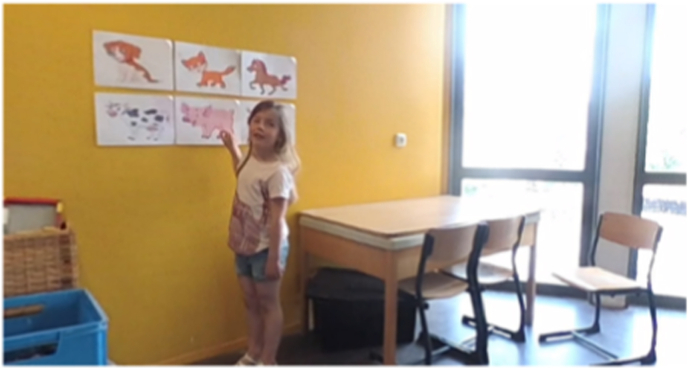
Prototype corridor.

**Fig. 4:**
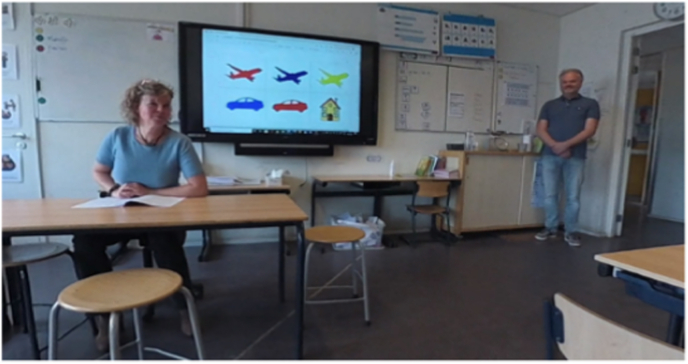
Prototype classroom.

During the ideation phase, psychologists and researchers were interviewed to define the requirements for a prototype that aligns with the CBT protocol. This led to the development of two distinct prototypes: one utilizing 2D 360° videos (see Fig. 2, Fig. 3, Fig. 4), selected for its realistic visual appearance and accessibility, as it can be easily viewed on a smartphone within an enclosed VR headset; and another built in Unity using 3D polygonal models on the Oculus Quest 2, offering greater interactivity and flexibility [27].

Each prototype featured three virtual environments (school playground, corridor, and classroom) and structured storylines designed to engage children in speaking exercises. The location of each storyline corresponds to the behavioral treatment. The child is required to respond verbally in class starting from step five. Therefore, the storylines for steps five through ten are set in the classroom environment. Due to the young age of the user and hardware limitations, the virtual environment is controlled through a dashboard by a remote supervisor. The usability and learnability of the prototypes were assessed using the System Usability Scale (SUS) [28] and semi-structured interviews. Both prototypes received satisfactory SUS scores: the 360° video-based prototype scored 70, and the Unity-based prototype scored 71, both exceeding the benchmark score of 68 [29,30].

Although the 360° video environment offers a higher degree of visual realism, it lacks the flexibility required for further customization and interactivity, therefore development continued using the Unity-based prototype. Based on feedback, further development in Unity focused on enhancing realism through lip synchronization, inverse kinematics to simulate natural human movement, and improved remote dashboard features.

**Fig. 5:**
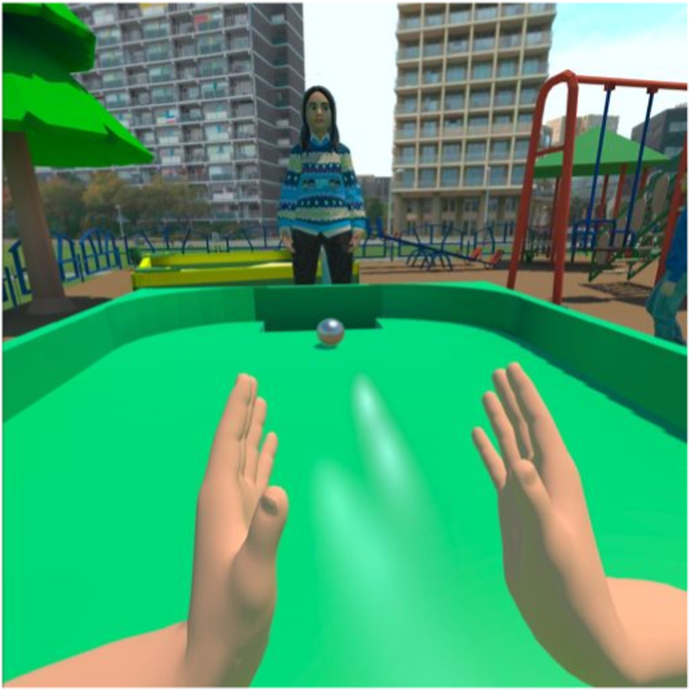
Step 1: Foosball playground.

**Fig. 6:**
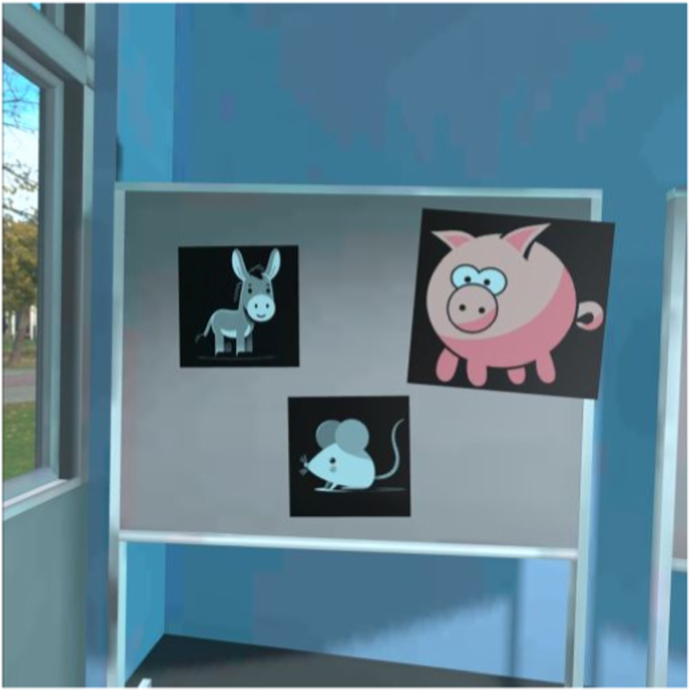
Step 3: Animal sounds Corridor.

**Fig. 7:**
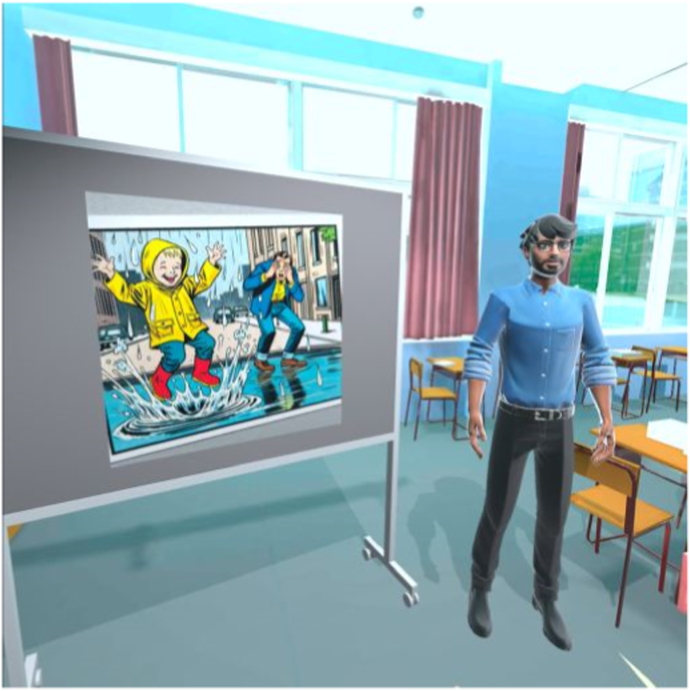
Step 6: Naming pictures Classroom.

The final version of Speaking at School-VR (see Fig. 5, Fig. 6, Fig. 7), delivered via the Meta Quest 3 headset, consists of 10 levels corresponding to the exposure hierarchy of individual treatment. The VR exercises have the same therapeutic goals practiced during in-person sessions at school. For example, making blowing sounds, vocalizations, repeating words, or forming short sentences. In this way, home-based VRET adds an intermediate step to the exposure hierarchy, enabling children to practice the same skills used in therapy within a safe and familiar environment and in the presence of a parent. This complementary structure is intended to support gradual progress, as the exercises may be easier to perform at home and before they are practiced in the school setting. Parents can further modify the difficulty level by increasing ambient noise and by adding up to six students to the virtual environment. There are four virtual teachers available representing various genders and ethnicities. Parents receive training to supervise their child's VR practice at home, ensuring structured and gradual exposure to speaking situations.

### 2.3 Assessments

Parents and teachers report on the ESM measures using the M-path smartphone application three times a week (Monday, Wednesday and Friday) for the entire study duration.

Supplementary clinical and standardized assessments will be administered online. Both parents are invited to fill out the questionnaires. If it is not feasible for both parents to participate, the parent who spends the most time with the child will be asked to complete the questionnaires. At baseline [T0], a comprehensive clinical assessment will be conducted to evaluate the primary study outcome, as well as relevant predictors, covariates, and potential confounding factors (see Table 2, for an overview). At the start of each new study phase [T1, T2, and T3], parents and teachers will report only on SM symptoms. At post-treatment [T4] and follow-up [T5], parents and teachers will report on both SM symptoms and confounding factors. A clinical interview with parents will be conducted during the pre-treatment, post-treatment, and follow-up assessments.

**Table 2:** Overview of assessments and informants.

Instrument	Variable	Moment of assessment					
		T0	T1	T2	T3	T4	T5
*Primary outcomes*							
Selective Mutism Questionnaire (SMQ-NL)	SM symptoms	P	P	P	P	P	P
Social Context of Speaking (SCS)	SM symptoms	T	T	T	T	T	T

*Secondary outcomes*							
Child Behavior Checklist (CBCL)	Behavioral and emotional problems	P				P	P
Teacher Report Form (TRF)	Behavioral and emotional problems	T				T	T
Structured Clinical Interview for DSM-5 disorders in children and adolescents (SCID-5 Junior)	DSM-5 classification of disorders	P				P^a^	P^a^
Fear Survey Schedule for Children Revised (FSSCR)	Anxiety symptoms	C				C	C
Parenting Burden Questionnaire (Opvoedingsbelangsting vragenlijst: OBVL)	Parenting burden	P				P	P
Clinical Global Impression Scale – Improvement	Severity of psychopathology		Th	Th	Th		
Child Observation Rating Scale	Overall Well-Being		C	C	C		
Visual Facial Anxiety Scale (VFAS)	Level of Anxiety		C	C	C		
Family Accommodation Scale (FASA)	Family accommodation	P				P	P
Virtual reality evaluation	Evaluation of VR		P	P	P	P	

*Predictors*							
Adult Self Report (ASR)	Adult problem behavior	P					
PABI	Parenting style	P					
Immersive tendencies – Parent version	Immersive tendencies	P					
Vineland-3-NL for parents	Adaptive functioning	P					
Vineland-3-NL for teachers	Adaptive functioning	T					
Demographic variables	Demographics	P					
Health care consumption questionnaire	Health care consumption	P					

T0: Baseline (2 weeks).

T1: Pre-treatment.

T2: After completion of step 5.

T3: After completion of step 10.

T4: Post-treatment.

T5: Follow-up (3 months).

P: parent.

T: teacher.

C: child.

Th: therapist.

a At T4 and T5 only the sections that met classification are assessed.

#### 2.3.1 Primary outcome: SM symptoms

*SCED*: For purpose of this study, the research team collaborated with experienced SM therapists and an expert on SCED in clinical settings to design a short 9-item questionnaire that is used as a frequent measurement for the SCED (see Appendices A and B). The questions on this frequent measure are assessed on a 5-point Likert scale. Parents and teachers are asked to report on the communicative and speaking behavior of the child in each specific week, three times a week (Monday, Wednesday and Friday) through a smartphone application, resulting in at least 117 time points.

*Clinical and standardized assessment*: Both parents fill out the Dutch translation of the Selective Mutism Questionnaire (SMQ-NL). The questionnaire contains 16 questions that are scored on a four-point Likert scale. The SMQ-NL has high reliability and good validity [31]. Teachers are asked to report on the Social Context of Speaking (SCS, see Appendix C).

#### 2.3.2 Secondary outcomes

*Emotional and behavioral problems:* will be assessed using the pre-school (ages 1,5–5) and school-aged (ages 6–18) versions of the Child Behavior Checklist (CBCL) and the Teacher Report Form (TRF) of the Achenbach System of Empirically Based Assessment (ASEBA). Both measures have a high reliability and validity [32,33].

*Anxiety*: The Dutch translation of the Fear Survey Schedule for Children Revised (FSSCR) will be used to measure symptoms of fear. The questionnaire measures the level of fear related symptoms and phobias in children across five different domains. The questionnaire has adequate reliability and validity [34].

*Psychiatric comorbidity*: the Structured Clinical Interview for DSM-5 disorder in children and adolescents (SCID-junior) will be administered. The SCID-junior is a reliable and valid instrument for the classification of DSM-5 disorders in children and adolescents [35].

*Parenting burden questionnaire*: the Dutch Parenting Stress Questionnaire will be filled out by parents (in Dutch translated as *Opvoedingsbelasting vragenlijst; OBVL)*. The OBVL contains five subscales and a total score. The OBVL has good reliability and adequate validity [36].

*Severity of psychopathology*: The Clinical Global Impression Scale (CGI) is a valid and reliable measure of severity of psychopathology across time [37]. The CGI has good validity and reliability estimates differ across populations [37].

*Family accommodation*: The Dutch translation of the Family Accommodation Scale – Anxiety (FASA) [38]. The FASA is a reliable and valid instrument for assessing family accommodation of childhood anxiety [39]. The FASA has also been validated for the Dutch population [40].

*VRET evaluation*: The time participants spent practicing the exercises will be monitored through the web interface which is used by the remote supervisor to control the virtual environment. The psychologist-researcher will plan a semi-structured interview with parents to document experiences of the VRET-and to ensure proper usage of the application. At the end of the study, parents and children are asked to share their experiences in a short interview.

After each session the following questionnaires will be filled out:

*Overall well-being*: The Childhood Outcome Rating Scale (CORS) will be used to track progress and well-being over the course of the treatment. After each session the therapist asks the child to report on four questions using a visual analogue scale. The CORS is a well-balanced self report measure between reliability and validity compared to longer measures [41].

*Anxiety*: Children are asked to rate their anxiety after each therapy session and VR session at home using the Visual Facial Anxiety Scale (VFAS) [42]. The VFAS is a valid measure of state anxiety.

#### 2.3.3 Covariates and predictors

*Parental behavioral and emotional problems*: Both parents are asked to complete the Adult Self-Report (ASR) to assess parental psychopathology. The ASR is an extension of the ASEBA scales CBCL and TRF. It provides a total score and scores for an internalizing and externalizing subscale. The Dutch self-report version has been proven to be a valid and reliable instrument to measure adult problem behavior [43].

*Parenting behavior*: The Parental Attitude and Behavior Index (PABI) consists of 20 scales that can be classified into three adaptive domains (warmth, structure and autonomy) and three maladaptive domains (hostility, control and overprotection). The PABI has demonstrated good reliability and validity [44].

*Immersive tendencies*: The Immersive Tendencies Questionnaire [45] is a valid and reliable instrument to assess immersion The questions were rephrased in such a way that parents can evaluate the immersive tendencies of their child.

*Communication*: The Vineland-3-NL will be used to assess adaptive functioning in the domain of communication and the subdomains receptive-, expressive- and written communication. The Vineland-3-NL is a valid and reliable instrument [46].

## 3 Statistical analyses

### 3.1 Sample size calculation

To establish the efficacy of a treatment, SCEDs involve the comparison of different treatment phases. For statistical analysis, each phase of the intervention needs at least five to 20 datapoints [34]. The study design has five phases, and it is ensured that each phase of the intervention will have at least five data points. SCED methodology provides substantial within-person statistical power due to the high number of repeated measurements per participant, ensuring that each individual case has sufficient data to detect meaningful within-person changes.

A power analysis was conducted using the Shiny app SCED Power Analysis [47]. A meta-analysis evaluating the efficacy of CBT for SM reported a large effect size (Hedges' adjusted g = 1.00 [0.62–1.39]) [48]. To detect an expected effect size of Cohen's d = 0.8 and an autocorrelation of 0.4, a sample size ranging from four to eight participants should provide sufficient power to establish an effect from baseline to intervention using the Permutation Distance Test [49]. The planned power analysis pertains to the aggregated, group-level effect, which will be estimated through a meta-analytic synthesis of the individual SCED p-values. The target sample size of *N* = 15 participants refers to the number of individual cases contributing to the pooled group effect.

Given that SM often occurs in young children the physical comfort of the headset might be compromised for children with smaller head sizes. To account for potentially higher dropout rates and missing data, our aim is to include 15 participants to ensure sufficient data for analysis.

#### 3.1.1 Primary outcomes

*SCED*: The ESM measure of SM symptoms is used to evaluate the primary outcome. In Single-Case Experimental Designs (SCEDs), visual analysis is the preferred starting point for assessing treatment effects. The ESM data are presented graphically to assess patterns in trend, level, and stability across and within intervention phases [50]. Additionally Tau-U will be reported [51]. Tau-U is a non-parametric effect size that was created for SCEDs reflecting the degree of non-overlap of observations between two phases. By incorporating both level changes and trend adjustments it is possible to control for baseline trends. Tau-U can be used for statistical testing and for meta-analytic purposes to establish a group effect of the intervention [51].

The Permutation Distance Test (PDT) [49] will be used to test the difference in medians between phases within an individual. The PDT tests the null hypothesis that the medians of the different phases are the same, while accounting for autocorrelation of the data.

*Standardized assessment:* To evaluate whether the change in the frequent assessment SM symptoms is reliable across the different study phases, a reliable change index (RCI) [52] will be computed for the SMQ and the Social Context of Speaking questionnaire. These questionnaires serve to validate the results obtained from the frequent measurements. To test for differences on a group level Friedman's analysis of variance (ANOVA) [53] will be used.

#### 3.1.2 Secondary outcomes

*Standardized assessment:* Overall well-being (CORS) and severity of psychopathology (CGI) will be plotted against time and will be subjected to visual inspection for upward and downward patterns. A Kendall's Tau correlation [54] will computed to evaluate whether there is a significant upward or downward trend in overall well-being or CGI scores during treatment.

The secondary outcomes changes in symptoms of anxiety (FSSCR), and psychological functioning (CBCL, TRF) between the baseline assessment, post-intervention assessment and follow-up assessment will be subjected to formal testing. These secondary outcomes are selected because they provide theoretically relevant validation and interpretation of the primary outcome measure. These outcomes will be evaluated by calculating an RCI. Friedman's ANOVA will be used to test for differences on a group level. Due to the risk of increased Type I error, appropriate correction methods for multiple comparisons where relevant (e.g., Benjamini–Hochberg) will be applied.

The remaining secondary measures, parenting burden (OBVL) and family accommodation (FASA), will be analyzed descriptively and treated as exploratory as these outcomes help to better understand the family system and how these factors may be associated with potential treatment effects. This approach allows us to maintain rigor in the interpretation of the primary endpoints, while using the secondary outcomes mainly to contextualize, validate, and deepen the understanding of the primary effects.

*Qualitative interview:* The qualitative data from the VR user experience evaluation will be examined using deductive thematic analysis to uncover and interpret key themes [55]. Qualitative interviews will complement the quantitative measures by providing insight into treatment acceptability and user experiences. These findings will help contextualize the quantitative outcomes and contribute to evaluating the feasibility of the intervention and the perceived role of VRET within the broader treatment. Feedback on the VRET application will also be collected to guide future improvements.

#### 3.1.3 Moderators and predictors

Demographic variables, psychiatric co-morbidity (SCID-5-NL), parental emotional and behavioral problems (ASR), parenting style (PABI), adaptive functioning (Vineland-3-NL), and immersive tendencies (ITQ-P) will be used to describe the participants.

#### 3.1.4 Handling of missing data

In single-case experimental designs (SCEDs), missing observations are not imputed for the purposes of visual analysis. Missing data points are displayed as gaps in the time series, allowing us to interpret potential patterns of missingness and to examine whether they may relate to the dependent variables or to contextual factors [56]. This is consistent with standard SCED methodology, in which transparency of the raw observation stream is essential for evaluating within-person change. For the statistical analyses, missing values will be handled differently. Because inferential SCED analyses require a complete time series within each participant, within-person imputation procedures will be applied.

## 4 Discussion

Behavioral therapy has been shown to be effective in the treatment of SM but can be lengthy and take up to an academic year, with many children having difficulties applying and practicing the learned skills in day-to-day speaking situations. Moreover, even after treatment, long-term psychological and communication problems may persist. The implementation of VRET at home offers a distinct advantage in the treatment of SM, providing children with a safe and engaging environment to become familiar with therapy exercises and to practice speaking more frequent within different school contexts from the comfort of their home. This may improve treatment outcomes, as the integration of VRET offers controlled and repeated exposure that to support the habituation and generalization processes that underlie effective exposure therapy. To the best of our knowledge, our study is the first to examine the efficacy of combining behavioral therapy with VRET at home as a complementary approach. Due to the novelty of the intervention a SCED was chosen, allowing for an in-depth examination of the treatment response on an individual level, while generating initial evidence of its feasibility and effectiveness.

### 4.1 Strengths and limitations

In addition to addressing some of the main the treatment challenges, the use of VRET at home gives parents a more central role in the treatment process, and might increase cooperation between parents and the therapist, especially since the behavioral therapy is conducted at school. For each step of the treatment protocol there is a range of exercises available that match the specific therapeutic goals of each treatment step. This enables parents to support their child throughout the treatment and helps them continue practicing in day-to-day situations as they become more familiar with the treatment principles and reinforce the progress made during therapy. This might enhance the effectiveness of the current treatment. Secondly, the use of VR amplifies the gamified elements of the treatment, which may, in turn increase participants’ motivation. Additionally, increasing the frequency and length of exposure sessions may help reduce treatment duration and improve treatment outcomes. This could potentially reduce the need for school visits.

Within the current study parents are involved as co-facilitators. To account for differences in parental involvement during the treatment and to better understand the role of family dynamics, we incorporated multiple assessments of family functioning to interpret the primary outcome in a broader contextual framework.

In contrast to randomized controlled trials, SCEDs do not provide the same degree of generalizability. However, the use of multiple SCEDs does allow for a detailed evaluation of an innovative, individualized VR treatment for SM, at both a group and individual level, while incorporating a multi-informant approach to strengthen the validity of the findings. The VRET module was designed as an adjunct to the current treatment. A potential limitation of this study is that the VRET module was offered alongside behavioral therapy at school, making it difficult to evaluate the effectiveness of the VRET module in isolation.

Nevertheless, the study will provide valuable insights into the feasibility of using VRET in a home setting. Additionally, our findings may provide initial clues about which client and family characteristics are positively associated with treatment gains. Lastly, participant's experiences and feedback will contribute to the further development and refinement of the VRET module for children with SM.

### 4.2 Implications for clinical practice

The aim of this study is to improve the treatment of children with SM by integrating VRET at home and thereby addressing several main challenges in the current treatment of SM. VRET at home may positively influence treatment effectiveness, By enabling more exposure within a shorter time frame and improving the generalization of the treatment effects. A shorter treatment duration may enable a larger number of children to receive care in the same amount of time and decrease logistical demands.

Implementing VRET at home may help reduce the burden on teachers, as parents can help their child with the therapy exercises at home. As a potential research direction, the feedback and user experiences of the current study may be used to develop a more comprehensive VRET-based treatment program. The VRET program could serve as an adjunct to treatment or offer an alternative to the individual sessions at school, for example during school holidays. If the current demonstrates that the VRET program can be used independently by parents, this may also open possibilities for the implementation of VRET during waiting periods, thereby offering some relief while children await treatment.

## CRediT authorship contribution statement

**Wendy van Vlerken:** Writing – original draft, Project administration, Methodology, Investigation, Conceptualization. **Jeroen S. Legerstee:** Writing – review & editing, Supervision. **Robert G. Belleman:** Writing – review & editing, Software. **Samantha Bouwmeester:** Writing – review & editing, Supervision, Methodology, Conceptualization. **Lynn F. Meester:** Writing – review & editing, Software, Methodology, Investigation. **Annelot Roorda:** Writing – review & editing, Software, Methodology, Investigation. **Ramón J.L. Lindauer:** Writing – review & editing, Supervision, Funding acquisition. **Elisabeth M.W.J. Utens:** Writing – review & editing, Supervision, Funding acquisition. **Luuk Stapersma:** Writing – review & editing, Supervision, Methodology, Investigation, Funding acquisition, Conceptualization.

## Available data and materials

Not applicable. This paper presents the study protocol and does not contain any data or results.

## Trial registration

ClinicalTrials.gov Identifier: NCT06882629.

## Ethical approval and consent to participate

The Medical Ethics Research Committee of the Amsterdam UMC has approved this trial (NL86645.018.24). This study will be conducted according to the Helsinki Declaration and its later amendments or comparable ethical standards. Informed written consents will be obtained from the parents or guardians of the participating children, and from the children (12+) themselves. This article does not contain any studies with animals performed by any of the authors.

## Consent for publication

All authors critically reviewed the manuscript for intellectual content. All authors have read and approved the manuscript for publication.

## Declaration of generative AI in scientific writing

During the preparation of this work the author(s) used ChatGPT (OpenAI) and Claude.ai (Anthropic) to improve the readability and clarity of the manuscript. After using this tool/service, the author(s) reviewed and edited the content as needed and take(s) full responsibility for the content of the publication.

## Funding

This research project is funded by the Vereniging van Nederlandse Gemeenten, Stichting Steun Emma Kinderziekenhuis, Stichting Burgerweeshuis Roomsch Catholiek Jongens Weeshuis and Cornelia-Stichting. The funding source had no role in the design of the study, and will not have any role in its execution, analysis, interpretation of the data, or decision to submit results.

## Declaration of competing interest

The authors declare the following financial interests/personal relationships which may be considered as potential competing interests: The authors declare that they have no known competing financial interests or personal relationships that could have appeared to influence the work reported in this paper.

## Data Availability

No data was used for the research described in the article.

## Footnotes

1 https://visualisationlab.science.uva.nl.
