# Supplementary material for: The efficacy of home-based virtual reality exposure therapy as an add-on to behavioral therapy for children with selective mutism: Protocol for a single-case experimental design
Source: Contemp Clin Trials Commun. 2026 Jan 16;50:101602. doi: 10.1016/j.conctc.2026.101602 (PMC12925134; doi:10.1016/j.conctc.2026.101602)
Supplement: Multimedia component 1 [file mmc1.docx]

| Appendix A. Frequent measurement parents | | | | | |  |
| --- | --- | --- | --- | --- | --- | --- |
| What behaviors do you see in your child in situations outside the home or with people other than family members? |  |  |  |  |  | |
|  | **Never** | **Rarely** | **Sometimes** | **Often** | **Always** | |
| 1. Communicates without words (but with gestures, eye contact, facial expressions) |  |  |  |  |  | |
| 1. Makes sounds |  |  |  |  |  | |
| 1. Uses one word (such as yes/no or other words) |  |  |  |  |  | |
| 1. Uses two words |  |  |  |  |  | |
| 1. Uses sentences of 3 words or more |  |  |  |  |  | |
| 1. Answers a question |  |  |  |  |  | |
| 1. Asks a question |  |  |  |  |  | |
| 1. Participates in conversations - individually/1-on-1 (with more than two sentences from the child) |  |  |  |  |  | |
| 1. Participates in conversations - in a group (with more than two sentences from the child) |  |  |  |  |  | |
