# Supplementary material for: The efficacy of home-based virtual reality exposure therapy as an add-on to behavioral therapy for children with selective mutism: Protocol for a single-case experimental design
Source: Contemp Clin Trials Commun. 2026 Jan 16;50:101602. doi: 10.1016/j.conctc.2026.101602 (PMC12925134; doi:10.1016/j.conctc.2026.101602)
Supplement: Multimedia component 3 [file mmc3.docx]

| Appendix C. Social Context of Speaking | | | | |
| --- | --- | --- | --- | --- |
| How often have you seen the following behavior in the child? |  |  |  |  |
|  | **Always** | **Often** | **Seldom** | **Never** |
| 1. Answers a question from their own teacher | | | | |
| 1. Answers a question from another teacher | | | | |
| 1. Answers a question from classmate | | | | |
| 1. Answers a question from other children | | | | |
| 1. Asks their teacher a question | | | | |
| 1. Asks another teacher a question | | | | |
| 1. Asks a classmate a question | | | | |
| 1. Asks another child a question | | | | |
| 1. Uses a whispering voice | | | | |
| 1. Uses speaking voice | | | | |
| 1. Always needs a warm-up game to start talking | | | | |
| 1. Uses functional speech | | | | |
| 1. Speaks with select group of classmates | | | | |
| 1. Speaks with all classmates | | | | |
| 1. Speaks spontaneously (says more than necessary) | | | | |
| 1. Speaks in class (when the whole class is listening) | | | | |
| 1. Speaks outside the classroom | | | | |
| 1. Continues talking when other children are around | | | | |
| 1. Does not talking interfere with school? | | | | |
